# Genetic Determinants of Coronary Artery Disease in Type 2 Diabetes Mellitus Among Asian Populations: A Meta-Analysis

**DOI:** 10.3390/medsci14010052

**Published:** 2026-01-21

**Authors:** Aida Kabibulatova, Kamilla Mussina, Joseph Almazan, Antonio Sarria-Santamera, Alessandro Salustri, Kuralay Atageldiyeva

**Affiliations:** 1 Department of Biomedical Sciences, Nazarbayev University School of Medicine, Astana 010000, Kazakhstan; aida.kabibulatova@nu.edu.kz (A.K.);; 2 Department of Medicine, Nazarbayev University School of Medicine, Astana 010000, Kazakhstanalessandro.salustri@nu.edu.kz (A.S.); 3 Department of Internal Medicine, Corporate Fund “University Medical Center”, Astana 010000, Kazakhstan

**Keywords:** type 2 diabetes mellitus, coronary artery disease, coronary heart disease, atherogenesis, atherosclerosis, genetic variants, genes, genome-wide association studies, single nucleotide polymorphisms, Asian population

## Abstract

**Background/Objectives**: Type 2 diabetes mellitus (T2DM) significantly elevates the risk of coronary artery disease (CAD), particularly in Asian populations where both conditions are epidemic. While shared genetic factors contribute to this comorbidity, evidence from Asian cohorts remains fragmented, with limited focus on population-specific variants. This meta-analysis synthesizes evidence on genetic variants associated with CAD risk in Asian patients with T2DM. **Methods**: We systematically searched several databases according to the PRISMA statement and checklist. Pooled odds ratios (ORs) with corresponding 95% confidence intervals (CIs) were calculated using random-effects models, with heterogeneity assessed via I^2^ and Cochran’s Q, and publication bias via funnel plots and Egger’s test. **Results**: In total, data on 11,268 subjects were reviewed, including 4668 cases and 6600 controls. Among 950 identified studies, 18 met eligibility criteria, and 14 studies provided sufficient data for the meta-analysis. The random-effects pooled estimate across all studied variants was not statistically significant (OR = 1.16 [95% CI: 0.68–2.00]; z = 0.56, *p* = 0.58). However, analysis of individual loci revealed gene-specific associations with CAD among this population: PCSK1 gene (OR = 2.12 [95% CI: 1.26–3.52]; *p* < 0.05; weight = 8.77%), GLP1R gene (OR = 2.25 [95% CI: 1.27–3.97]; *p* < 0.01; weight = 8.62%). ADIPOQ gene (OR = 8.00 [95% CI: 2.34–27.14]; *p* < 0.01; weight = 6.35%). Several genes were associated with an elevated risk of CAD: PCSK1 gene (OR = 2.12 [95% CI: 1.26–3.52]; *p* < 0.05; weight = 8.77%), GLP1R gene (OR = 2.25 [95% CI: 1.27–3.97]; *p* < 0.01; weight = 8.62%) and ADIPOQ gene (OR = 8.00 [95% CI: 2.34–27.14]; *p* < 0.01; weight = 6.35%). Several genes were associated with possible protective effects: ACE gene (OR = 0.41 [95% CI: 0.23–0.73]; *p* < 0.01; weight = 8.57%), Q192R gene (OR = 0.20 [95% CI: 0.08–0.52]; *p* < 0.001; weight = 7.41%). Heterogeneity was substantial (τ^2^ = 0.78; I^2^ = 81.95%; Q (13) = 64.67, *p* < 0.001). **Conclusions**: This first meta-analysis of genetic variants associated with CAD in Asian populations with T2DM identified specific locus-level associations implicating lipid metabolism, incretin signaling, and oxidative stress pathways. The lack of a significant pooled effect, alongside high heterogeneity, underscores the complexity and population-specific nature of this genetic architecture. These findings suggest that effective precision risk stratification may depend more on specific variants than on a broad polygenic signal, highlighting the need for further research in a larger, distinct sample size.

## 1. Introduction

Diabetes mellitus has emerged as one of the most pressing non-communicable diseases of the 21st century, contributing substantially to global morbidity and mortality. Diabetes has reached global epidemic proportions, according to the International Diabetes Federation (IDF) evaluations. To date, 589 million adults are living with it, and the number is projected to reach 853 million people by 2050 [1]. Asian regions, like East and Southeast Asia, are among the largest contributors in these proportions, accounting for 247.6 million diabetic patients [1,2,3]. Type 2 diabetes mellitus (T2DM) is the most prevalent type, according to the Global Burden of Disease (GBD) study, representing 96.0% of all cases worldwide in 2021 [4]. The burden of T2DM is rising and is projected to grow, with the steepest increases observed in the Asian region [5,6,7,8].

T2DM is a well-known independent risk factor for atherosclerosis and is currently considered a cardiovascular disease itself [9]. Comprehensive epidemiological data indicates that individuals with T2DM are most likely to die from coronary artery disease (CAD) with cardiovascular disease accounting for about 75–80% of all fatalities in the diabetic population [10,11]. Moreover, patients with T2DM have a higher prevalence of CAD compared to those without this condition [12,13,14], and cardiovascular diseases remain the main leading causes of death among all diabetes-related complications. This startling mortality rate emphasizes how vital it is to comprehend the complex relationship between both diseases and indicates the need for early diagnosis and accurate prognosis as well as efficient preventive strategies.

There are several pathogenetic pathways that converge to accelerate atherosclerosis in T2DM patients. Chronic hyperglycemia drives the non-enzymatic attachment of sugars to circulating and tissue-resident proteins, lipids, and nucleic acids, forming a diverse array of advanced glycation end products (AGEs) [15,16,17]. AGEs stiffen the vessel wall and trap low-density lipoprotein (LDL), making it more susceptible to oxidation and activating pro-atherogenic signaling cascades [18,19,20]. Simultaneously, hyperglycemia and insulin resistance increase oxygen form production, depleting nitric oxide availability and triggering oxidative stress [21,22,23]. The dysfunctional endothelium becomes pro-thrombotic, expressed through elevated fibrinogen, von Willebrand factor, and plasminogen activator inhibitor-1, leading to resistant fibrin clots [24,25,26,27,28,29,30]. An atherogenic lipid profile, characterized by elevated triglycerides, reduced high-density lipoprotein cholesterol, and abundant small, dense LDL particles, further accelerates atherogenesis [31,32,33,34].

However, classical risk factors and established pathophysiologic mechanisms do not fully account for the heightened CAD burden in T2DM. A substantial genetic component contributes significantly, as both diseases demonstrate high heritability, approximately 58% for CAD and 72% for T2DM, and genome-wide association studies (GWAS) have identified hundreds of susceptibility loci, with evidence of pleiotropic genes affecting both conditions [35,36,37,38,39,40,41,42]. Despite these advances, most genetic research focuses on European populations, severely limiting applicability to Asian cohorts [43,44,45]. CAD-specific genetic variants in T2DM patients remain understudied, particularly in Asian populations.

This meta-analysis synthesizes evidence on genetic determinants of CAD in T2DM across Asian cohorts.

## 2. Materials and Methods

### 2.1. Protocol and Registration

This meta-analysis followed the Preferred Reporting Items for Systematic Reviews and Meta-Analyses (PRISMA) 2020 guidelines [46]. The study protocol was registered in the International Prospective Register of Systematic Reviews (PROSPERO) under the registration number CRD420252163559.

### 2.2. Eligibility Criteria

Studies were selected according to the Population, Intervention/Exposure, Comparison, Outcome, and Study design framework (PICOS). The inclusion criteria were as follows: individuals older than 18 years, of Asian population with a confirmed diagnosis of T2DM based on a fasting blood glucose level of over 126 mg/dL (7 mmol/L); oral glucose tolerance test level higher than 200 mg/dL (11.1 mmol/L) after 2 h; level of glycated hemoglobin over 6.5%. Studies were included if they used a case–control or cross-sectional design that allowed comparison between T2DM patients with confirmed CAD (cases) and T2DM patients without CAD (controls).

The CAD in case group was defined using established clinical and diagnostic criteria aligned with American Heart Association (AHA) and European Society of Cardiology (ESC), including angiographically confirmed coronary stenosis of 50% or greater in major vessels, documented myocardial infarction according to standardized definitions, coronary revascularization procedures such as percutaneous coronary intervention or coronary artery bypass grafting, and acute coronary syndromes [47,48,49,50]. Secondary outcomes were defined to assess the phenotypic severity and premature CAD. CAD severity was assessed using standardized angiographic classification systems where data were available: for studies reporting detailed angiographic data, the SYNTAX score was applied [51]; if the study included categorization by the vessels’ obstruction, Coronary Artery Disease Reporting Data System (CAD-RADS) and risk stratification were used [52,53]. Premature CAD was defined as an atherosclerotic disease occurring at the at ≤55 years of age in men and ≤65 years of age in women, consistent with major clinical guidelines [54].

The genetic exposures of interest include various types of genetic variants; specifically single nucleotide polymorphisms (SNP) identified via GWAS arrays or sequencing.

Studies were excluded if they met any of the following conditions: did not present the summary statistics or effect estimate and its precision in terms of an odds ratio (OR) with the corresponding 95% confidence interval; non-original research formats, such as editorials, reviews, case reports, and conference abstract.

### 2.3. Search Strategy and Study Selection

A literature search was conducted using the Scopus, Web of Science, PubMed and Ovid MEDLINE databases. The search strategies applied various combinations of medical subject heading terms and keywords. Queries for searches were customized for each database according to its distinct characteristics. The search technique encompassed collecting English-language research published from 1 January 2001 to October 2025. The collected publications were organized and managed utilizing Zotero, version 7.0.30 (Corporation for Digital Scholarship, Vienna, VA, USA) reference management software.

### 2.4. Data Extraction and Quality Assessment

Data were extracted by two researchers independently (A.K. and K.M.). All disagreements were resolved by discussion to reach a final consensus. Discrepancies that could not be resolved by discussion were adjudicated by a third reviewer (K.A.). The information extracted from each eligible study was as follows: first author’s name, year of publication, ethnicity/geographic region of the study population, participants with and without CAD and T2DM, the number of participants, mean age and gender distribution of cases and controls, number of SNPs (rsID), OR (95% CI), and evidence of Hardy–Weinberg equilibrium (HWE). The OR was interpreted as a prevalence OR for cross-sectional studies and a disease OR for case–control studies. These measures were pooled under the standard genetic meta-analysis assumption that CAD is a stable outcome, allowing the prevalence OR to approximate the risk OR.

The quality of the study was evaluated using the Joanna Briggs Institute (JBI) critical appraisal tools specific to the study design. The JBI tool was chosen for its suitability to assess observational genetic association studies. For case–control studies, quality appraisal focused on clarity of inclusion criteria, appropriateness of control selection, assessment of confounding, and validity of statistical analysis [55]. Two reviewers (A.K. and K.M.) conducted an independent quality appraisal, with discrepancies resolved by consensus or consultation with a third reviewer (K.A.).

### 2.5. Statistical Analysis

The association of genetic variants with CAD among T2DM patients was calculated by pooled odds ratios (ORs) with corresponding 95% confidence intervals (CIs). Odds ratios from both case–control and cross-sectional study designs were synthesized together, a standard meta-analytic approach for genetic association studies, under the assumption that the CAD phenotype was consistently defined. In this meta-analysis, the natural logarithm of the odds ratio (log OR) was employed as the primary effect size metric for pooling genetic associations under the model reported as the primary analysis in each study (e.g., co-dominant, dominant, recessive, and multiplicative models). This approach was chosen to minimize post hoc data transformation and respect the original authors’ analytical design. For studies reporting multiple models, the most common model across the literature was identified from a pre-screening of published reports and selected to ensure consistency. The Z test was used to determine the significance of the pooled OR (*p* < 0.05) was considered statistically significant. A random-effects model with restricted maximum likelihood (REML) estimation was applied to account for potential heterogeneity among studies. Heterogeneity was also evaluated using the Cochran’s Q statistic (*p* ≤ 0.10 considered significant) and quantified with the I^2^ statistic with thresholds of 25%, 50%, and 75% representing low, moderate, and high heterogeneity, respectively. To assess data quality, genotype data from control groups were evaluated for deviations from Hardy–Weinberg equilibrium (HWE) using a Chi-square test (*p* < 0.05 considered a significant deviation). The potential influence of any study with significant HWE deviation was examined in a sensitivity analysis. For genes with multiple reported polymorphisms (e.g., ADIPOQ), each variant was assessed individually to account for potential differences in function and linkage disequilibrium, thereby avoiding conflation of distinct genetic effects. These variants were treated as independent genetic markers and analyzed separately; they were not pooled into a composite gene-level estimate.

Publication bias was evaluated through funnel plots and statistically using Egger’s regression test and Begg’s test. Funnel plot asymmetry was examined visually. Sensitivity analyses were performed by sequentially omitting individual studies to assess the stability of the results. All statistical analyses were conducted using STATA 16.1 version (STATA Corporation, College Station, TX, USA).

Superficial text editing (grammar, spelling, and punctuation) was performed using Grammarly (Grammarly Inc., San-Francisco, CA, USA).

## 3. Results

**Figure 1:**
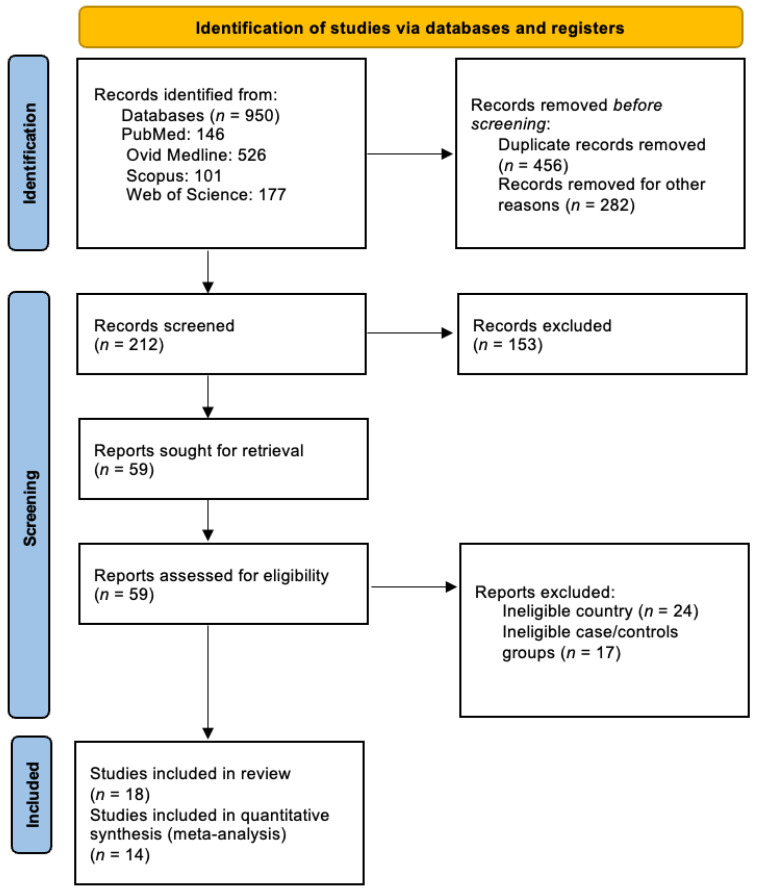
PRISMA flow diagram.

A total of 950 records were identified through database searches (PubMed = 146; Ovid Medline = 526; Scopus = 101; Web of Science = 177). After removing duplicates and a thorough full-text screening, 18 eligible studies were included. The detailed study selection process is illustrated in Figure 1.

In total, data on 11,268 subjects were reviewed, including 4668 cases and 6600 controls. Among 18 eligible studies [56,57,58,59,60,61,62,63,64,65,66,67,68,69,70,71,72,73] 14 studies were included for the meta-analysis, as they had sufficient quantitative data (Table 1). Most studies were case–control in design, with two cross-sectional studies. The majority originated from East Asia (China and Japan), while several were carried out in Middle Eastern populations (Iran, Saudi Arabia, and the United Arab Emirates). Patients with familial hypercholesterolemia, an inherited autosomal dominant disorder caused by mutations in genes like LDLR, APOB, or PCSK9, were not included in any of the studies selected for the metanalysis. The average age of participants ranged between 52 and 63 years, and women accounted for 35% to 57% of the study samples. The studies varied considerably in size, with cases ranging from 100 to 560 and controls from 72 to 2424 participants. The included studies evaluated a broad range of genetic polymorphisms. Variants within the *ADIPOQ* gene (3q27–28) were the most frequently examined, including rs266729, G276T, 276G/T and rs2241766. Other loci of interest comprised *ACE* (rs4646994, 17q23.3–4), *APOE* (rs429358, 19q13.2), *CNR1* (G1359A, chr 6), *PON1* (Q192R), *GLP-1R* (rs4714210, 6p21.2), *SIRT1* (rs16924934, 10q21.3), *STK11* (rs12977689, 19p13.3), *PCSK1* (rs3811951, 5q15–q21), *PLXDC2* (rs12219125), and *PSRC1–CELSR2* (rs599839). Polymorphisms within *ADIPOQ*, *ACE*, *APOE* and *PON1* were most consistently associated with elevated risk of CAD, while *GIP*, *PCSK1* and *PSRC1–CELSR2* variants were more frequent among T2DM patients without CAD.

**Figure 2:**
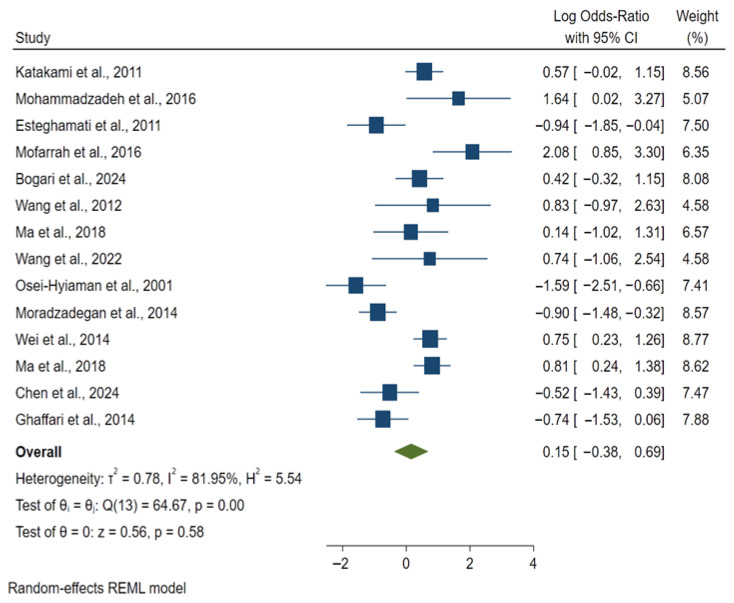
Forest plot of selected studies. Blue squares represent the log odds ratios with 95% CI for individual studies; green diamond represents the pooled effect estimate from the random-effects model [56,57,59,60,62,64,65,66,67,69,70,71,72,73].

Individual study estimates displayed considerable variability, with several indicating a potential significant association with the disease (log OR < 0) alongside others suggesting null or adverse associations; notably, the CIs for most studies overlapped the null value (log OR = 0), precluding statistical significance at the study level. The random-effects model yielded a pooled OR of 0.15 (95% CI: −0.38–0.69), suggesting a trend toward a protective effect. However, this was not statistically significant (z = 0.56, *p* = 0.58). Heterogeneity across studies was substantial (τ^2^ = 0.78; I^2^ = 81.95%; H^2^ = 5.54), Cochran’s Q test indicated significance (Q (13) = 64.67, *p* < 0.001). Individual study contributions in the forest plot (Figure 2) varied by direction and precision, with high-weight studies (8–9%) including Wei et al., 2014 [60]; *PCSK1* gene; log OR = 0.75 [95% CI: 0.23, 1.26]; OR = 2.12 [95% CI: 1.26, 3.52]; *p* < 0.05; weight = 8.77%) and Ma et al., 2018 [57] *GLP1R*; log OR = 0.81 [95% CI: 0.24, 1.38]; OR = 2.25 [95% CI: 1.27, 3.97]; *p* < 0.01; weight = 8.62%), both indicating significant associations with increased risk, offset by protective effects in Moradzadegan et al., 2014 [62] *ACE*; log OR = −0.90 [95% CI: −1.48, −0.32]; OR = 0.41 [95% CI: 0.23, 0.73]; *p* < 0.01; weight = 8.57%). Outlying low-weight studies with wide CIs included Mofarrah et al., 2016 [69] *ADIPOQ*; log OR = 2.08 [95% CI: 0.85, 3.30]; OR = 8.00 [95% CI: 2.34, 27.14]; *p* < 0.01; weight = 6.35%; strong risk) and Mohammadzadeh et al., 2016 [65] *ADIPOQ;* log OR = 1.64 [95% CI: 0.02, 3.27]; OR = 5.15 [95% CI: 1.02, 26.32]; *p* = 0.05; borderline risk; weight = 5.07%; limited by small sample size), alongside significant protection in Osei-Hyiaman et al., 2001 [72] *PON1* (Q192R); log OR = −1.59 [95% CI: −2.51, −0.66]; OR = 0.20 [95% CI: 0.08, 0.52]; *p* < 0.001; weight = 7.41%). These opposing signals contributed to the non-significant pooled estimate.

**Figure 3:**
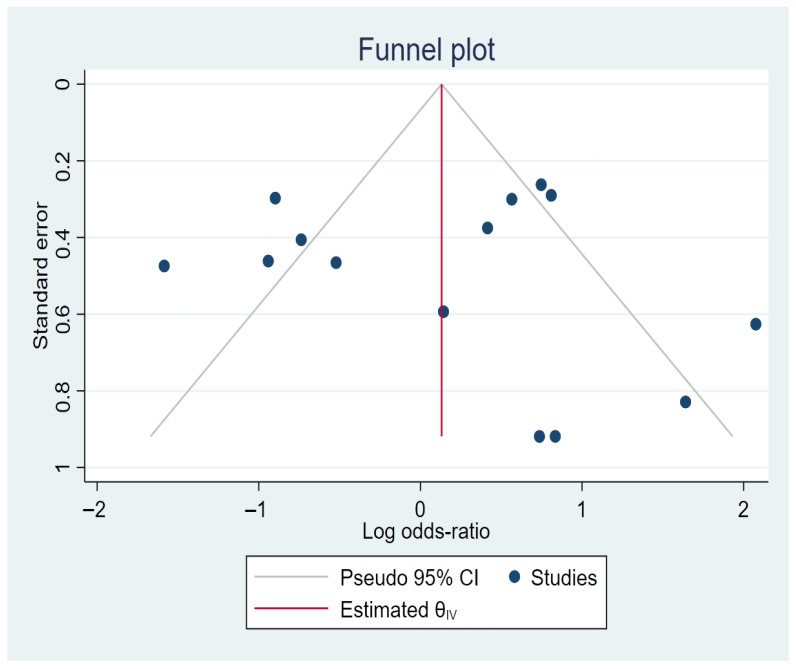
Funnel plot of selected studies.

Potential publication bias was explored using a funnel plot Figure 3. The funnel plot analysis revealed a generally symmetrical distribution of studies around the pooled effect, with only a limited number of points slightly beyond confidence intervals. Studies located outside the funnel boundaries are more likely attributable to heterogeneity of studies. This analysis revealed no evident pattern of absent studies or distortion in the funnel plot, indicating a lack of significant publication bias and reinforcing the validity of the meta-analytic estimate; however, the influence of small-study effects cannot be entirely ruled out.

## 4. Discussion

This meta-analysis provides a comprehensive synthesis of genetic variants linked to the development of CAD in T2DM patients within Asian populations. Our findings illustrate the complex variations of genetic susceptibility and the diverse characteristics of coronary risk among diabetic patients in various Asian subgroups. Across the chosen 14 studies, examining different genetic polymorphisms provided substantial insight into the genetic component of CAD susceptibility among Asian T2DM patients. Variants of the ADIPOQ gene have been identified as the most investigated and consistently linked to the increased risk of CAD [74,75]. The established roles of the adiponectin pathway in insulin sensitization, anti-inflammatory processes, and cardiovascular protection support the biological plausibility of these associations. The mechanisms underlying risk signals in ADIPOQ variants align with reduced adiponectin levels, which contribute to pro-inflammatory and insulin-resistant conditions that enhance atherogenesis [76]. ACE rs4646994 presumably enhances vascular remodeling and thrombosis through elevated angiotensin II levels. APOE rs429358 is associated with elevated risk of coronary complications and adverse lipid profiles PON1 Q192R may affect the oxidative modification of lipoproteins [77,78]. In contrast, the protective associations for PCSK1 and PSRC1–CELSR2 align with improved prohormone processing and lipid regulation, respectively, whereas GIP-linked loci correspond with enhanced incretin action.

During the analysis, several polymorphisms showed higher frequency in TD2M without CAD, suggesting potential protective properties towards CAD. In some studies, the GLP-1R rs4714210 variant demonstrates protective effects, with GG genotype displaying a markedly decreased risk of CAD, even after optimizing for conventional risk variables [79]. This protection likely indicates enhanced GLP-1 receptor activation, which confers coronary protection via various mechanisms, including improved endothelial function, decreased inflammation, and increased insulin sensitivity. Enhanced GLP-1R activation may improve coronary outcomes by restoring endothelial homeostasis through cyclic adenosine monophosphate/protein kinase A—dependent endothelial nitric oxide synthase phosphorylation, increased nitric oxide bioavailability, and reduced oxidative stress. This results in improved flow-mediated dilation, coronary flow reserve, and myocardial perfusion [80]. Concurrently, GLP-1R agonism reduces vascular inflammation by decreasing macrophage infiltration and pro-inflammatory cytokines and reinstates endothelial redox signaling, thereby collectively decreasing atherosclerotic plaque progression and instability. The biological outcomes correspond with cardiovascular outcome trials indicating a reduction in major adverse cardiovascular events associated with GLP-1 receptor agonists, thereby supporting a translational connection between endothelial and anti-inflammatory advantages and event reduction. Recent meta-analyses provide evidence of significant reductions in major adverse cardiovascular events, stroke, and cardiovascular mortality at the class level, highlighting the cardioprotective effects of GLP-1 receptor agonists that extend beyond mere glycemic control [81]. This protection may arise because PCSK1 encodes prohormone convertase 1/3, which activates hormones by processing proinsulin to insulin and proglucagon to GLP-1. Therefore, any protective effect from rs3811951 is likely associated with altered prohormone processing, islet biology, and metabolic phenotypes [82]. This may also be attributed to findings from genome-wide meta-analysis indicating that rs599839 at chromosome 1p13.3 is significantly associated with LDL-cholesterol, suggesting the involvement of this locus in lipid regulation, which aligns with cardio protection through enhanced lipoprotein profiles [83]. The cardioprotective mechanism of rs599839(G) is closely associated with its effect on lipid homeostasis, a crucial pathway that is dysregulated in T2DM, leading to foam cell formation and plaque instability [84].

The minimal variability highlights how consistent these signals are across different studies, which may be due to common environmental modifiers in Asian cohorts, like high-carbohydrate diets and insulin resistance brought on by urbanization, which enhance genetic resilience without superseding it [85,86,87,88,89,90]. However, given that recent analyses show that shared genetic architectures between T2DM and CAD are still underpowered in smaller regional studies, the non-significant pooled OR emphasizes the need for larger, multi-ancestry GWAS to detect subtle polygenic effects.

Limitations of this analysis must be considered. A primary limitation is a non-significant pooled estimate. The lack of an overall significant association represents a serious constraint, indicating that the results do not support a unified genetic model. Consequently, the nominally significant subgroup findings should not be generalized; they require rigorous independent confirmation in larger, well-defined groups.

A substantial heterogeneity observed in our meta-analysis (I^2^ = 81.95%), is a probable cause of the non-significant pooled estimate. The observed inconsistency is likely attributable to divergent CAD diagnostic criteria, the substantial genetic and environmental diversity within the broadly defined Asian study populations, and technical discrepancies in sample sizes and genotyping protocols across the included studies.

Visual inspection of the funnel suggested approximate symmetry, and formal tests did not indicate significant publication bias. However, the small number of studies limits the power of these assessments. Phenotypic heterogeneity in CAD definitions across studies, ranging from angiographically confirmed stenosis (≥50%) to clinical diagnoses and revascularization procedures, may have contributed to inconsistent associations.

The studies included in the metanalysis did not provide systematic data on the glycemic control, presence of hypercholesterolemia, and smoking habits, which are well known risk factors for CAD. Although lifestyle can increase the risk for cardiovascular disease, the aim of this study was to look specifically into the genetic variants associated with CAD in T2DM among Asian population.

## 5. Conclusions

This first meta-analysis investigating genetic underpinnings of CAD in Asian individuals with T2DM identified specific locus-level associations but found no significant aggregate genetic effect across variants. The substantial heterogeneity and non-significant pooled estimate underscore the complexity and likely population-specific nature of this genetic risk. Consequently, these findings suggest that precision medicine approaches may need to target specific population-relevant variants rather than relying on a broad polygenic signal from these loci. Future research must prioritize larger, genetically homogeneous cohorts to validate these candidate gene associations and elucidate their interaction with environmental factors.

## Figures and Tables

**Table 1:** General characteristics of the selected studies.

Author/Year	Population	Mean Age (±SD)	Females (%)	SNP	Cases (n)	Controls (n)	CAD Definitions		
							1	2	3
Wang et al., 2012 [71]	Chinese Han	57 (±10)	51.7	G1359A	259	191	+		
Tong et al., 2013 [58] *	Chinese	61 (±8)	44.6	rs266729	560	550	+		
Katakami et al., 2011 [59] *	Japanese	55 (±8)	38.9	G276T	213	2424		+	
Ma et al., 2018 [57]	Chinese Han	62 (±10)	39.3	rs4714210	394	217	+		
Ma et al., 2018 [67]	Chinese Han	63 (±10)	43.7	rs8078510	390	276	+		
Wei et al., 2014 [60]	Chinese Han	63 (±10)	45.4	rs3811951	425	258	+		
Wang et al., 2022 [56]	Chinese Han	63 (±8)	45.9	rs16924934	297	195	+		
Ma et al., 2017 [61]	Chinese Han	63 (±10)	41.8	rs12977689	288	159	+		
Osei-Hyiaman et al., 2001 [72]	Chinese	63 (±7)	40.7	PON 1	201	231	+		
Chen et al., 2024 [73]	Chinese	60 (±9)	35.7	rs429358	378	351	+		
Ghaffari et al., 2014 [64]	Iran	52 (±6)	56.49	rs2794521	151	157	+		
Moradzadegan et al., 2014 [62]	Iran	59 (±8)	51.76	rs4646994	141	369	+		
Lei et al., 2012 [68]	Chinese	60 (±12)	45.72	rs4646994	220	318			+
Mohammadzadeh et al., 2016 [65]	Iran	55 (±9)	53.5	276G/T	100	100	+		
Azzam et al., 2019 [63]	UAE	61 (±11)	57.49	rs12219125	160	245		+	
Esteghamati et al., 2011 [70]	Iran	56 (±11)	52.28	276G/T	114	127	+		
Mofarrah et al., 2016 [69]	Iran	58 (±8)	49.11	rs2241766	152	72	+		
Bogari et al., 2024 [66]	Saudi Arabia	60 (±6)	39.78	rs599839	225	360	+		

* cross-sectional studies; CAD definitions: **1**—coronary artery disease diagnostic criteria, where patients with CAD was defined as subjects who had angiographic evidence of stenosis of 50% or greater in at least one major coronary artery, confirmed by coronary angiography; **2**—coronary artery disease diagnostic criteria, where patients were with diagnosis of myocardial infarction, that was performed by cardiologists based on the clinical symptoms, characteristic ECG changes, cardiac enzyme levels, and the findings in coronary angiography and/or echocardiography; or ICD-9 diagnostic codes 410–414; **3**—coronary artery disease diagnostic criteria, where patients were with angiographic evidence of 75% stenosis of at least one major coronary artery, or a history of prior angioplasty or coronary artery bypass surgery, or myocardial infarction by history validated by electrocardiographic changes; SNP—single nucleotide polymorphisms; SD—standard deviation; UAE—United Arab Emirates. + indicates that the corresponding method was used by the authors of the original article.

## Data Availability

No new data were created or analyzed in this study.

## Abbreviations

The following abbreviations are used in this manuscript:

AGEs	advanced glycation end products
CAD	coronary artery disease
CIs	95% confidence intervals
GWAS	genome-wide association studies
HWE	Hardy–Weinberg equilibrium
IDF	International Diabetes Federation
JBI	Joanna Briggs Institute
LDL	low-density lipoprotein
NO	nitric oxide
OR	odds ratio
PICOS	Population, Intervention/Exposure, Comparison, Outcome, and Study
PRISMA	Systematic Reviews and Meta-Analyses
PROSPERO	Prospective Register of Systematic Reviews
REML	restricted maximum likelihood
SNP	single nucleotide polymorphisms
T2DM	type 2 diabetes mellitus
